# Utilization of Waste Glass in Autoclaved Silica–Lime Materials

**DOI:** 10.3390/ma15020549

**Published:** 2022-01-12

**Authors:** Katarzyna Borek, Przemysław Czapik

**Affiliations:** Faculty of Civil Engineering and Architecture, Kielce University of Technology, Al. Tysiąclecia Państwa Polskiego 7, 25-314 Kielce, Poland; p.czapik@tu.kielce.pl

**Keywords:** waste glass aggregate, container glass, stained glass, autoclaved bricks, autoclaving, SEM, XRD

## Abstract

This paper aims to investigate the possibility of using waste glass of different colours as a complete substitute for quartz sand in autoclaved silica–lime samples. On the one hand, this increases the possibility of recycling waste glass; on the other hand, it allows obtaining autoclaved materials with better properties. In this research, reference samples with quartz sand (R) and white (WG), brown (BG), and green (GG) waste container glass were made. Parameters such as compressive strength, bulk density, and water absorption were examined on all samples. The samples were examined using a scanning electron microscope with an energy dispersive spectroscopy detector (SEM/EDS) and subjected to X-ray diffraction (XRD) analysis. The WG samples showed 187% higher compressive strength, BG by 159%, and GG by 134% compared to sample R. In comparison to the reference sample, volumetric density was 16.8% lower for sample WG, 13.2% lower for BG, and 7.1% lower for GG. Water absorption increased as bulk density decreased. The WG sample achieved the highest water absorption value, 15.84%. An X-ray diffraction analysis confirmed the presence of calcite, portlandite, and tobermorite phases. Depending on the silica aggregate used, there were differences in phase composition linked to compressive strength. Hydrated calcium silicates with varying crystallisation degrees were visible in the microstructure image.

## 1. Introduction

The overall amount of waste generated within the EU-27 (27 Member States of the European Union) amounted to 2317 million tonnes in 2018. This represents 5.2 tonnes of waste per one EU27 inhabitant [1]. Different economic and household activities contribute to waste generation in the following sectors: construction (36% of the total amount in 2018), mining and quarrying (26.2%), processing (10.6%), wastewater services (9.9%), households (8.2%), and other (9.1%). Waste packaging has a significant share in the waste quantity generated by households. Between 2008 and 2018, the main packaging waste components in the EU included paper and cardboard (a total of 31.8 million tonnes in 2018), followed by plastic and glass (14.8 million tonnes of plastic and 14.5 million tonnes of glass in 2018) [2]. In 2018, the total amount of packaging waste generated in the EU27 was estimated to be 77.7 million tonnes, representing a slight increase of 0.3% compared to the previous year. That was mainly due to an increase in the quantity of packaging made of glass (+3.1% compared to 2017), but also plastic (+1.7%) and paper and cardboard (+1.0%). In 2008, the total per capita waste originating from packaging materials within the EU averaged 161.6 kg. In the decade following on from 2008, that figure increased by 12.4 kg to a record of 174.0 kg per capita waste (from 67.8 kg in Croatia to 227.5 kg per capita waste in Germany) [2].

Container glass, such as bottles and jars, can be recycled almost completely and with no time restrictions, without compromising its quality or purity [3]. The Glass Packaging Institute, representing the US glass packaging industry, has determined that a tonne of recycled glass saves over a tonne of natural resources. Furthermore, recycling six tonnes of container glass results in one tonne less carbon emissions, which has significant environmental benefits [4].

There are two approaches adopted in the market for glass cullet recycling, i.e., open and closed-loop recycling. The closed-loop approach focuses on material reuse. However, due to high temperature and relatively long silica melting time, glass production consumes considerable amounts of energy [5]. Whilst, theoretically, glass waste can be fully recycled into a new glass product, minor impurities with a distinct composition or colour are not accepted in the glassmaking process. Furthermore, the complex sorting of fine glass particles makes recycling more expensive [6]. More efficient options for waste glass reuse are therefore being sought. The answer to that is the open-loop approach. This recycling mode usually involves the one-time use of waste glass as an ingredient in materials with long service life, e.g., wall construction materials. Up to now, waste glass has been utilised for the production of concrete [7,8,9,10], mortars [11,12,13,14], ceramic components [15,16], autoclaved concrete [17], or sand–lime products [18,19,20], to name a few. Crushed waste glass can be used as a partial substitute for cement/lime, sand, and/or coarse aggregate. Utilised in that manner, the glass requires no heat treatment, which is beneficial in reducing energy consumption. As a result, the processing of glass waste is greatly simplified, and its utilisation share is increasing along with the demand for construction materials. In the era of construction industry development with the growing demand for residential buildings, the increase in production, e.g., masonry wall materials, is a normal effect. According to GUS (Statistics Poland) of 2018 [21], bricks made of cellular concrete, ceramics, and silicate are the most popular materials. Two of the above also have specific production methods in common. These include the autoclaved materials: autoclaved cellular concrete and sand–lime products.

The sand–lime products, also known as silicates or “white bricks”, are made from three basic materials: sand (92%), lime (8%), and water. Quartz sand as a crystalline silica carrier acts as the reactive aggregate, which reacts with hydrated quicklime, a binder [22]. In the autoclaving process, the lime, as a carrier of Ca^2+^ cations and OH^−^ anions, after hydration, reacts with silicate ions which originate from the dissolution of SiO_2_, forming products included in the group of hydrated calcium silicates [23]. A solid bond between the unreacted aggregate and the binder matrix is thus created. As the production process uses only natural raw materials with no chemical additives and low energy consumption, autoclaved materials are fully sustainable.

In the literature are known modifications of silica–lime products based on various types of waste [20,24,25,26,27,28,29,30]. Waste materials have been often utilised as substitutes for aggregate in conventional products. Studies have demonstrated that ternary blends of lime, sand, and recycled waste glass had higher compressive strength and lower density than the reference sample. The increase in those parameters was commensurate with the amount of substitute in the blends [18].

The use of amorphous glass sand as a substitute for crystalline quartz sand (90% by weight) was investigated by A. Stępień et al. [19]. As for the physical and chemical properties, the compressive strength of silica–lime samples with glass addition was improved (20 MPa with glass addition vs 6.5 MPa in the reference samples). With the increase in the percentage of the above additive, the bulk density decreased slightly (1.7 kg/dm^3^–>1.65 kg/dm^3^), while the moisture content increased (from 0.5% to 2.1%). The share of glass sand in silicate products is therefore considered beneficial.

Since the glass industry has a diversified and complicated production structure, it is estimated that the number of developed glass types (compositions) currently amounts to 70–80,000. The range of this industry includes 20–30,000 varieties of products. The variety of manufacturing methods and applications and chemical and granulometric compositions of glass products have resulted in the need to divide the glass industry into four basic sectors: construction glass, household and lighting glass, technical glass and container glass [31]. Soda-lime glass is used for the majority of glass packaging products. The chemical composition of the glass used for glass packaging production also depends on the colour. Stained glass can be achieved by adding appropriate metal oxides to the glass mass. Table 1 presents the chemical compositions of glass by colours.

There are many studies on the influence of colour and glass type on the physicochemical properties of construction materials. While testing cement blends, Dyer and Dhir [34] found that mortar containing 10% white and green cullet had approximately 27 MPa and 20 MPa higher compressive strength after 28 days than the reference mortar. The mortar containing 10% powdered amber glass (a different name for brown glass) achieved only approximately 2 MPa higher compressive strength than the reference mortar. Karamberi and Moutsatsou [33] used standard soda-lime glass from recycled packaging in their study. The authors observed that the compressive strength of mortar containing 25% powdered white glass was about 34.5% higher than that containing 25% brown glass. They attributed this discrepancy to the basic elements colouring the glass particles and claimed that their presence would cause a chemical reaction responsible for compressive strength development. Mirzahosseini and Riding [35] reported that mortar containing green glass cullet below 25 µm in size had approximately 3% higher compressive strength after 91 days than white glass. The authors concluded that the solubility of silicon and aluminium in green glass is high, which results in an increased pozzolanic reaction at the high pH of the cement matrix. Furthermore, Bignozzi et al. [36] explained that the glass dissolution rate increases with the increase in the PbO + Na_2_O content and with the decrease of both glassy forms SiO_2_ + Al_2_O_3_ and CaO + MgO glass stabilisers. Glass with a dusty fraction containing a higher quantity of modifiers and a lower amount of glassy substances and glass stabilisers does not favour the pozzolanic reaction. As a result, the authors stated that the mortars containing ground soda-lime glass showed approximately 4% higher compressive strength after 90 days than mortars made of crystal glass. Al-Zubaid et al. [37] reported that concrete with neon glass revealed approx. 9.5% higher compressive strength than concrete using green waste glass for 13% cement replacement. They attributed that effect to a higher share of SiO_2_ (68.2%) and CaO (22.6%) in the neon glass compared to the green glass. On the other hand, Zimmer and Braganca [38] stated that the chemical compositions of clear and stained glass powders are very similar and assumed that the finished products would not demonstrate varying properties due to the addition of glass with different colours. It should also be noted that when glass is used in cement products curing under normal conditions, apart from the favourable pozzolanic reaction, the so-called alkali-silica reaction may occur, which is a destructive process [11,12,13]. The research conducted so far shows that the colour of the glass may also affect its course. Whether the pozzolanic or alkali-silica reaction occurs is primarily determined by the fragmentation of the material.

Until now, most of the studies show that glass colour, due to the variation in chemical composition, can significantly impact the development of compressive strength in mortar and concrete. So far, most studies have been based on the use of waste glass as a partial replacement for sand while increasing the percentage of binder in the blend. However, there are no studies in which the only aggregate is waste glass, and the mass amount of binder is the same as in the standard blend. The primary objective of this study is to verify the possibility of utilizing waste container glass as a total substitute for quartz sand in sand–lime products. Given the discrepancies resulting from the literature review, another objective is to identify the effect of the used glass colour on the physical and mechanical properties of autoclaved bricks.

## 2. Materials and Methods

### 2.1. Materials Characterization

#### 2.1.1. Lime

Ground quicklime is a product obtained by crushing and grinding lumps of quicklime. The lime used in this study comes from Sitkówka Trzuskawica A CRH Company (Poland). The chemical composition presented in Table 2 meets the requirements for quicklime used to produce lime and silica products. The bulk density of the lime is 0.79 kg/dm^3^. Reactivity 600 °C ≤ 2.0 min. A positive soundness test proves that the lime is not overburned and does not contain excessive magnesium oxide (MgO) with dolomite admixture. Lime sieve analysis with 2 mm mesh = 100% passing, 0.2 mm mesh ≥ 97% passing by weight and 0.09 mm mesh ≥ 90% passing by weight complies with PN-EN 459-2:2010 [39].

#### 2.1.2. Quartz Sand

Quartz sand with a continuous grain size curve without a clear dominance of any grain fraction is considered suitable for producing sand–lime products. The grain size composition of quartz sand was determined following PN-EN 933-1:2012 [40]. The percentages of individual fractions with specifications for quartz sand and the sand used in this study are listed in Table 3.

The quality of quartz sand also depends on its chemical composition. Sand types with a silica content of at least 80% are considered helpful in producing sand–lime products. The remainder is composed of elements listed in Table 4, where their mass percentage in the tested sand sample is given. The quartz sand used in the study meets the quantitative specifications for the chemical composition. The grains are irregular in shape with a medium roundness degree. The density of the sand is 2650 kg/m^3^.

#### 2.1.3. Water

According to PN-EN 1008:2004 [41], potable water, which is not subject to additional quality tests, is readily suitable for the production of silicate products. Such water was therefore used as an ingredient in the silica–lime mix.

#### 2.1.4. Container Glass

Two types of container glass from two sources were used in the study. Those were narrow-bore glass from green and brown bottles and wide-bore glass from clear (white) jars. Glass cullet was obtained from the solid input material by mechanical crushing. The grain sizing was adjusted to the distribution of the quartz sand fraction as the aggregate was replaced at a 1:1 weight ratio.

**Figure 1:**
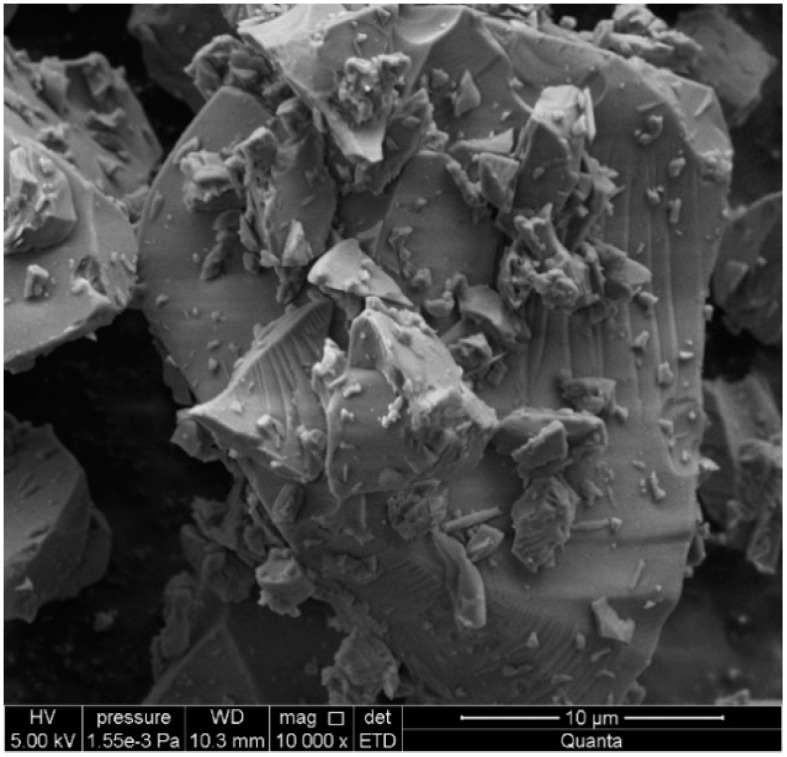
Microstructure of packaging glass.

**Figure 2:**
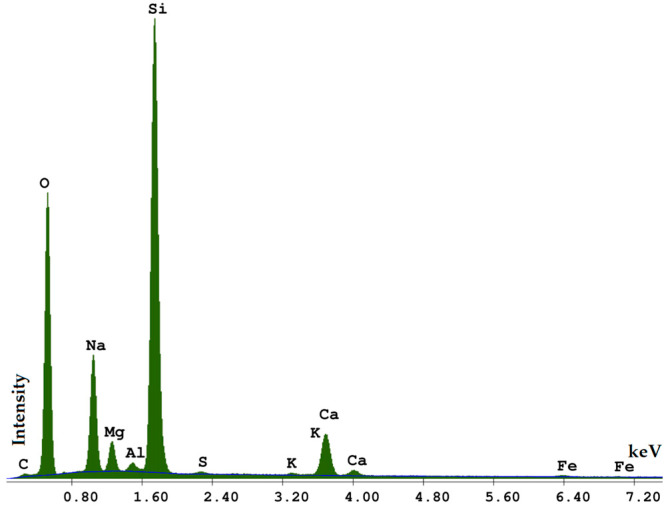
EDS of packaging glass.

Irregular and sharp-edged shapes of the waste glass particles are found in the microstructural image (Figure 1). The EDS (Energy Dispersive Spectroscopy) elemental analysis (Figure 2) reveals the content of Si, Na, Mg, Al, S, K, Ca in the material. The specific density of the glass types with respect to colour is as follows: white glass 2479 kg/m^3^, green glass 2494 kg/m^3^, and brown glass 2495 kg/m^3^.

### 2.2. Preparation of Silica–Lime Samples with the Addition of Coloured Container Glass

**Figure 3:**
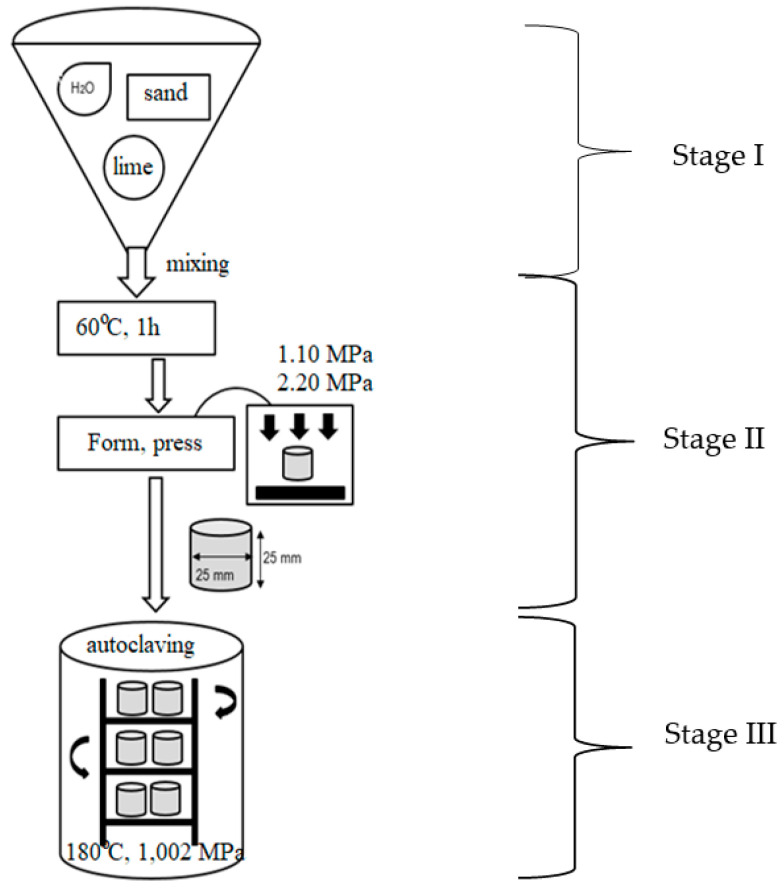
Scheme for the preparation of silica–lime samples involving packaging glass.

The quartz sand, constituting 92% of the sand–lime mixture, was replaced by three types of waste glass (clear/white glass—WG, green glass—GG, and brown glass—BG) (Table 5). The container glass cullet replaced the sand fractions in a 1:1 ratio. Three series of samples were prepared. Silicate, in which quartz sand was the aggregate, was used as a reference sample—R. The same preparation scheme was followed each time (Figure 3).

**Figure 4:**
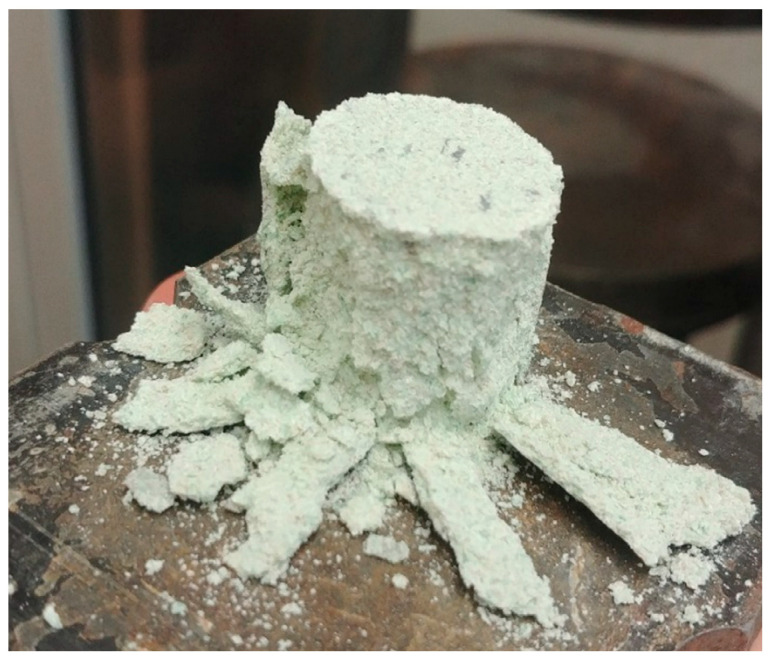
Broken silica–lime sample with GG after compressive strength test.

First, the individual dry ingredients were weighed. After mixing, water was added to obtain mass with a 6–8% moisture content. The mass was then placed in a sealed glass vessel and heated at 65 °C. After 1 h, the mass was cooled to ambient temperature, and the ingredients were mixed again. The next step was to form cylindrical samples with a height and diameter of 25 mm. The mass was placed in a steel mould and subjected to two-stage pressing at 10 MPa and 20 MPa with venting between the stages. The last stage, autoclaving, was divided into three phases (Figure 4). In the first stage, the samples were heated in an autoclave to a temperature of 180 °C for 2.5 h. When the assumed temperature and steam pressure were obtained, the second stage began, in which the samples were autoclaved at 180 °C under a saturated steam pressure of 1.002 MPa for 8 h. The third stage lasted 12 h and involved cooling the samples to ambient temperature.

### 2.3. Testing Methods

The experimental samples were tested for physical and mechanical parameters. For each recipe, 12 cylindrical 2.5 × 2.5 cm samples were made. Six of these samples were used in bulk density and water absorption test. Compressive strength tests were carried out on the remaining six samples. Single pieces formed after the compressive strength test were used to investigate the phase composition and microstructure of the samples. The tests were performed on six samples each time. Mean values of the obtained results, including the standard deviation, are shown in individual charts. The samples prepared for testing compressive strength, volumetric density, and water absorption were stored in laboratory conditions at room temperature. The SEM analysis of the microstructure was carried out on crumb specimens prepared as a result of compressive strength tests. The methodology of conducted research is presented in Table 6.

## 3. Results and Discussion

**Figure 5:**
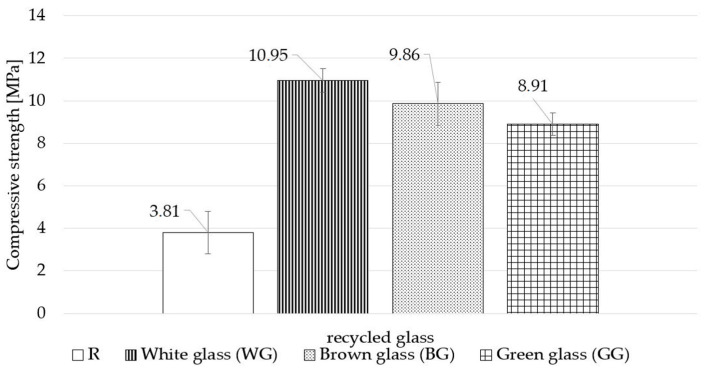
Compressive strength test results.

Figure 4 shows the broken silica–lime GG sample after compressive strength test. The compressive strength results of the silica–lime products modified with different colour glass are shown in Figure 5. Replacing quartz sand with waste glass, regardless of the colour, results in a compressive strength increase of the samples prepared, compared to the samples of standard composition. The highest increase of 10.95 MPa was recorded for the WG sample, which was 187% above R, followed by the BG sample with 159% increase (9.86 MPa) above R. The lowest increase, of 8.91 MPa (134% above R) was recorded for the GG sample. The compressive strength of the WG samples was 11.1% higher than that of the GG samples. The use of waste glass as a substitute for quartz sand was also found to have improved the compressive strength in the study of Stępień and all [44]. The hydration temperature of lime with the increasing share of WG was lowered, hence it can be deducted that hydration of lime during the production of autoclaved samples provided adequate chemical bonds, and as a result strength of samples increased. It should be also noted that calcium hydroxide shows retrograde solubility with respect to the temperature, so it is possible that at this lower temperature more calcium ions were released upon dissolution and available for the reaction to form hydrates.

**Figure 6:**
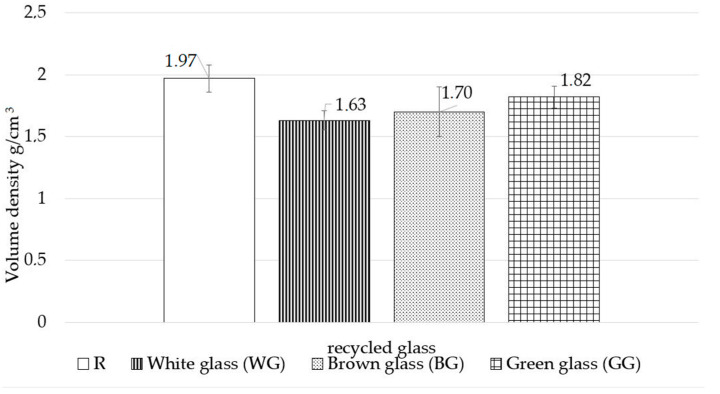
Volume density results.

**Figure 7:**
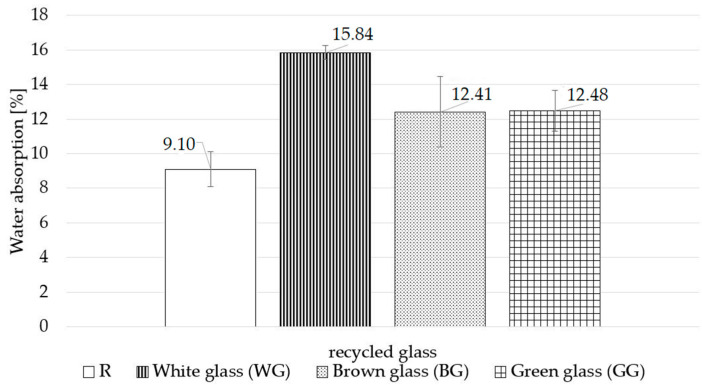
Results of water absorption.

**Figure 8:**
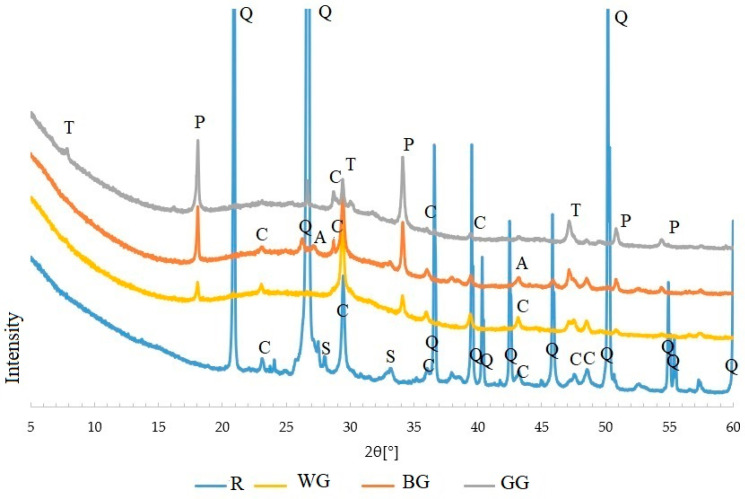
X-ray-pattern of WG, BG, and GG samples (symbols: A—aragonite, C—calcite, P—portlandite, S—spurrite, T—tobermorite, Q—quartz).

**Figure 9:**
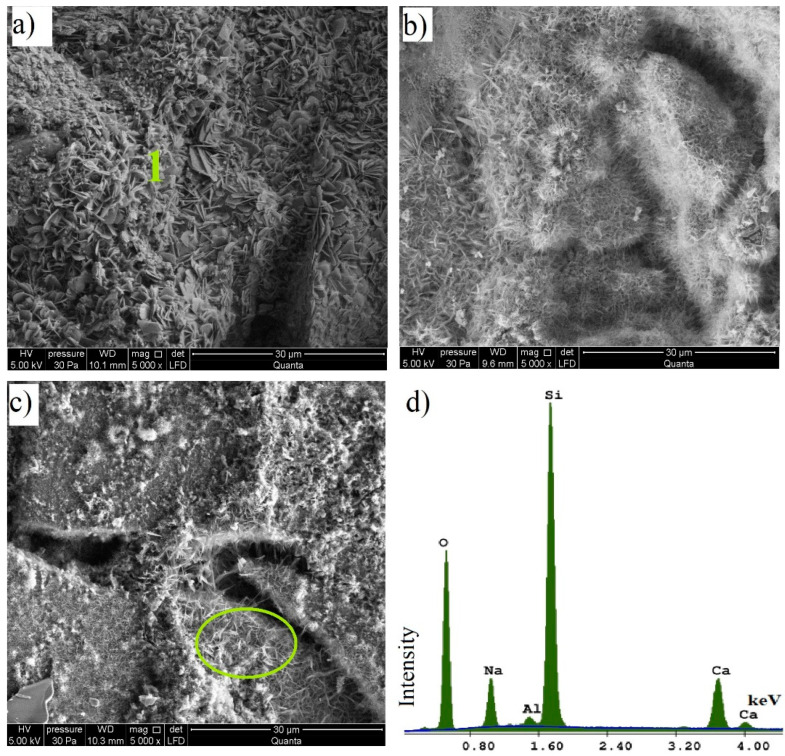
Microstructure of samples (**a**) WG, (**b**) BG, (**c**) GG, and (**d**) EDS analysis at point 1 in (**a**).

Figure 6 shows the volumetric density and Figure 7 the water absorption. Volumetric density examination proves that the replacement of quartz sand by waste container glass of different colours positively affects the volumetric density results of the autoclaved samples. The glass colour is also important in this case since the lowest volumetric density was achieved by the WG sample (16.8% decrease). The second result of 1.70 g/cm^3^ had the BG sample, representing a decrease of 13.3% relative to the R sample. The lowest decrease was recorded for the GG sample—1.82 g/cm^3^ (7.1% decrease). The WG and GG samples demonstrate the most significant differences in volumetric density. The volumetric density of the GG sample is 10.4% above that of the WG sample. These differences can be attributed to the density of the aggregate used in the sample, as the density of the green glass cullet is 0.6% higher than that of the white glass cullet. The differences in the densities of the studied samples may be due to their different chemical properties. They may form other phases and microstructures, the determination of which is the subject of the XRD and SEM/EDS studies presented in Figure 8 and Figure 9, respectively. The phases formed can affect the porosity of the autoclaved samples.

In each case studied, replacing quartz sand with waste glass increases water absorption. The brown and green glass samples show comparable values of 12.41% (BG) and 12.48% (GG). The WG samples recorded the most significant increase in water absorption (15.84%) compared to the standard composition. The increased water absorption in the studied case should be attributed not as much to the material substituting the quartz sand but to the increased open porosity. In the performed study, there is an evident relationship between the compressive strength and bulk density and water absorption due to the colour of used waste glass. The WG samples show the highest compressive strength and absorption, yet the lowest density relative to the BG and GG samples. The GG samples, in turn, have the lowest compressive strength and water absorption and the highest density compared to the WG and BG ones.

The phase composition of the silica–lime products shown in Figure 8 indicates that calcium hydroxide (portlandite) formed in all samples with glass aggregate. Peaks typical of calcite are also found, confirming portlandite carbonation. Carbonation may cause the lack of cohesion of the sample subjected to compression, Figure 4. In the analysed samples, hydrated calcium silicates appear as hydration products. A peak characteristic of tobermorite (5–10° 2θ) reaches the highest intensity in GG sample. In the other samples, this peak does not occur. In all samples containing glass, however, a raised background is visible, which may be caused by the presence of unreacted glass and the low degree of structural order of formed hydrated calcium silicates. Their structure may be similar to the C-S-H phase structure formed in natural conditions [45]. This confirms the observation that in the autoclaving process, the presence of reactive silica in the amorphous state increases the amount of the C-S-H phase, but at the same time, the transformation of this phase into a more stable form is hindered [46].

When samples with different glass colours were tested, the main differences in the diffractograms were the intensities of the portlandite peaks. Significantly, for samples for which higher compressive strengths were achieved (Figure 5), correspondingly lower intensities of peaks of portlandite were found. The decrease in intensity of these peaks can be related to a higher degree of portlandite reactivity, which indicates a more efficient autoclaving process. However, portlandite also reacted in the previously mentioned carbonation process, which is also associated with a decrease in its content.

Peaks of unreacted quartz were also detected in the GG sample, which, together with high portlandite peaks, indicate the lowest reactivity degree of the green glass cullet. Despite the similarities in the chemical composition of white, brown, and green waste glass, the results obtained do not confirm the assumptions of Zimmer and Braganca [38]. Silica–lime samples with the above-mentioned glass colours show variable properties.

No evident changes that could indicate the presence of hydrated calcium silicates were detected on the reference sample X-ray pattern. Instead, the most intense peaks from calcite were observed, which may indicate a significant degree of carbonation. This may be the reason for this sample’s low compressive strength performance, where the calcium binder has carbonated instead of reacting with the silica [47,48]. In addition, due to quartz sand, very intense quartz peaks are observed in this sample.

Figure 9 shows SEM images of silica–lime samples containing the WG, BG, and GG at a 5000× magnification. Hydrothermal synthesis products with a lamellar pattern formed between glass-derived silica and lime were found in each analysed sample. These products cover the entire surface of the samples. The WG and BG samples show densely packed formations surrounding the glass aggregate. In the GG sample, the synthesis products were formed in the spaces between and on the surface of the glass aggregate; however, they are much less dense/packed and reveal a lower crystallisation degree. The products formed in the studied samples resemble hydrated calcium silicates typical of the standard sand–lime products [44]. Based on the microstructure observation, it can be concluded that their development degree varies according to the waste glass colour used. In the WG sample (Figure 9a), the hydrated lime silicates took the form of small plates with rounded edges, resembling clover leaves in shape, which are closely spaced and arranged at different angles. These silicates can be identified as tobermorite (Figure 9d), whose high content may be related to the increased compressive strength of this sample [46,49,50,51]. In this sample, however, a lot of pores occurring between the produced plates are observed. There are more of these pores than in other samples tested and they may be the reason for increased water absorption results (Figure 7). According to Černý et al. [49], the high strength achieved by autoclaved materials may be due to the presence of tobermorite, which can be increased by using suitable raw materials. However, this effect may be diminished when the raw materials used increase the porosity of the material, as may be the case with fluidised bed ash in the zone around the ash grains. In the case of white glass (WG), the pores visible in the SEM images did not cause such an effect. As in the study by Černý et al. [49], the use of container glass contributed to a significant increase in compressive strength. In the present study, the increase in strength of the material is observed, which is also linked to the formation of tobermorite during the autoclaving process [50,51].

In the BG sample (Figure 9b), the hydrated lime silicates resemble narrow plates with an irregular arrangement (grass shape). The analysed GG sample (Figure 9c) is mostly covered with a spongy formation typical of the C-S-H phase. Its higher level of crystallization was found in a small area visible in the marked circle.

## 4. Conclusions

Based on the findings of the study, the following conclusions can be drawn:

- a complete substitution of quartz sand with glass waste aggregate in silica–lime products is possible and has a beneficial effect on selected physical properties of the finished products;
- the use of different colours of waste glass has a variable impact on the physical properties of the autoclaved silica–lime products;
- regardless of the glass colour used, the tested samples demonstrate a significant increase in the compressive strength of finished products; the use of white waste glass (WG) results in a 184% increase in strength with a 159% increase for brown glass (BG) and 134% increase for green glass, compared to the samples with the standard composition;
- regardless of the glass colour used, the samples demonstrate a decrease in volumetric density and an increase in water absorption;
- in samples containing waste glass, the study reveals the formation of portlandite and calcite is the main product, regardless of the colour of the glass, additionally, based on the SEM/EDS analysis, a tobermorite phase was found;
- the microstructure image shows the formation of phases typical of autoclaved sand–lime products, the colour of the glass affects to a varying extent the crystallisation degree of hydrated calcium silicates;
- white glass aggregate is the most reactive and has the strongest effect on the properties of silica–lime products. Green glass proved to be the least reactive.

## Figures and Tables

**Table 1:** Chemical compositions of glass by colours (content in % by weight) [32,33].

	White Glass			Brown Glass			Green Glass		
	Jin, W., Meyer, C., Baxter, S.	Karamberi A., Moutsatsou A.	Poland/HS “Pollena Czechy”	Jin, W., Meyer, C., Baxter, S.	Karamberi A., Moutsatsou A.	Poland/HS “Ujście”	Jin, W., Meyer, C., Baxter, S.	Karamberi A., Moutsatsou A.	Poland/HS “Ujście”
SiO_2_	73.20–73.50	70.65	71.20	71.90–72.40	71.20	71.30	71.30	70.5	71.00
Na_2_O + K_2_O	13.60–14.10	13.80	13.00	13.80–14.40	13.75	14.00	13.10	13.40	14.00
CaO + MgO	10.70–10.80	13.15	14.50	11.60	12.95	11.40	12.20	12.90	11.20
Al_2_O_3_	1.70–1.90	1.75	1.55	1.70–1.80	1.90	2.50	2.20	1.80	2.50
SO_3_	0.20–0.24	0.45	-	0.12–0.14	0.30	-	0.05	0.25	-
Fe_2_O_3_	0.04–0.05	0.45	0.03	0.30	0.35	0.30	0.56	0.45	0.60
Cr_2_O_3_	-	-	-	0.01	0.06	-	0.43	0.25	0.20

**Table 2:** Chemical composition of lime.

Chemical Composition of Lime	Requirements for Lime	Lime Used in the Study
CaO + MgO	≥90%	≥91%
MgO	≤5%	≤2.0%
CO_2_	≤4%	≤3.0%
SO_3_	≤2%	≤0.50%

**Table 3:** The percentages of individual fractions with specifications for quartz sand and of the sand used in this study.

Individual Fractions	Requirements for Sand	Sand Used in the Study
2.5–0.5 mm	<30%	2.5–0.5 mm 19%
0.5–0.05 mm	≥65%	0.5–0.05 mm 81%

**Table 4:** Weight % of elements in quartz sand.

Element	Weight (%)	Atomic (%)	Net Int.
C	3.73	6.43	53.28
O	41.79	54.14	1976.71
Mg	0.44	0.38	34.33
Al	4.50	3.46	363.93
Si	46.60	34.39	3655.03
K	0.71	0.38	32.18
Fe	2.23	0.83	30.50

**Table 5:** Quantitative summary of the silica–lime mixture in each series [% mass].

	Reference (R)	White Glass (WG)	Brown Glass (BG)	Green Glass (GG)
Glass	0	92	92	92
Quartz sand	92	0	0	0
Lime	8	8	8	8

**Table 6:** The methodology of conducted research.

**Compressive strength**	Laboratory conditions at room temperature Equipment: Controls 50-C9030 hydraulic press, Manchester, Barcelona PN-EN 772-1+A1:2015-10 [42]
**Volumetric density**	Hydrostatic method
**Water absorption**	PN-EN 772-21:2011 [43]
**Microstructure**	Scanning electron microscopy (SEM) Equipment: SEM-type, Quanta 250 FEG, Brno, Czech Republic Signals collected by secondary electron (SE) detectors The microstructures of samples were examined under low vacuum conditions without sputtering pre-treatment, using 5-kV voltage. EDS (Energy Dispersive Spectroscope—EDAX)
**Phase composition**	X-ray diffractometry (XRD) method X-ray analysis was performed using the DSH (Deby-Scherrer-Hull) powder method. The samples were previously ground by hand in a mortar. The tests were carried out in the range of 5–70° (2θ) at the registration speed of 0.0005° (2θ)/s. Equipment: Empyrean, PANALYTICAL, Almelo, Netherlands with a Cu lamp.

## Data Availability

No new data were created or analyzed in this study. Data sharing is not applicable to this article.
